# *Drosophila* as a Genetic Model System to Study Organismal Energy Metabolism

**DOI:** 10.3390/biom15050652

**Published:** 2025-05-01

**Authors:** Arely V. Diaz, Izel Tekin, Tânia Reis

**Affiliations:** Division of Endocrinology, Metabolism and Diabetes, Department of Medicine, University of Colorado Anschutz Medical Campus, Aurora, CO 80045, USA

**Keywords:** Drosophila, metabolism, obesity

## Abstract

Metabolism is the essential process by which an organism converts nutrients into energy to fuel growth, development, and repair. Metabolism at the level of a multicellular, multi-organ animal is inherently more complex than metabolism at the single-cell level. Indeed, each organ also must maintain its own homeostasis to function. At all three scales, homeostasis is a defining feature: as energy sources and energetic demands wax and wane, the system must be robust. While disruption of organismal energy homeostasis can be manifested in different ways in humans, obesity (defined as excess body fat) is an increasingly common outcome of metabolic imbalance. Here we will discuss the genetic basis of metabolic dysfunction that underlies obesity. We focus on what we are learning from *Drosophila melanogaster* as a model organism to explore and dissect genetic causes of metabolic dysfunction in the context of a whole organism.

## 1. Metabolic Dysfunction and Obesity

Obesity, a result of metabolic imbalance, is increasingly prevalent in human populations. According to the World Health Organization, in 2022, 43% of adults are classified as overweight and 16% of adults are obese worldwide (https://www.who.int/news-room/fact-sheets/detail/obesity-and-overweight, accessed on 21 April 2025). Considering the strong association between obesity and metabolic syndrome and a confluence of predisposing factors, including environment, diet, and genetic background, it has been challenging to identify causes and effective treatments for obesity. Twin studies estimate that genetic background contributes to 40–90% of obesity occurrence [1,2]. One of the first mouse models of inherited obesity derived from knockout of the *obese* (*ob*) gene [3]. The *ob* ortholog in humans is leptin, a protein hormone that is produced in adipocytes and regulates appetite and satiety (reviewed in [4,5]). A loss-of-function *leptin* mutation was among the first discovered monogenic causes of human obesity [6]. Other known monogenic forms of obesity affect the gene encoding the leptin receptor [7] or melanocortin, another neuropeptide that controls food intake [8,9]. Known monogenic causes of obesity are few and thought to be rare. Indeed, nearly all known monogenic sources of human obesity arise from mutations in what subsequently emerged as a leptin-melanocortin signaling pathway that controls food intake [10,11,12,13,14,15,16,17,18]. Genetic studies like these were key to developing insights into the contributions of genetic background to metabolic dysfunction and obesity.

Later, genome-wide association studies (GWAS) identified hundreds of alleles at other loci that correlate with the prevalence of obesity [19]. Despite this progress, identifying specific genetic causes of obesity has been challenging due to the complexities of gene-environment interactions in the regulation of metabolic homeostasis. Monogenic and polygenic contributors to metabolic disorders influence an individual’s metabolic response to environmental factors. When such complexity is at play, simpler experimental systems offer many advantages. In the lab we can control for many of the contributing factors, particularly environment and genetic background, while manipulating gene function to narrow down the gene networks and pathways that are involved in the regulation of energy homeostasis. When studying the genes and pathways that regulate response to the environment and integrate signals across a variety of tissues and organs, a robust genetic toolbox capable of manipulating gene function in specific tissues, plus the ability to control an organism’s environment, are powerful features of an experimental system. Over the past few decades, with approaches like these, *Drosophila melanogaster* has emerged as such a system.

## 2. From Humans to Flies

*Drosophila melanogaster* is a powerful animal model to study metabolic homeostasis, not only due to its power as a robust genetic model system to study human disease [20,21], but also due to the presence of analogous tissues, organs, and cell types that mimic human physiology [22,23]. For example, the *Drosophila* fat body and oenocytes are counterparts of human adipose tissue, liver, and immune system. In the fat body, levels of circulating sugars are balanced between glycogen stores and intake from diet via a functional ortholog of glucagon and insulin-like peptides [24,25,26].

**Figure 1:**
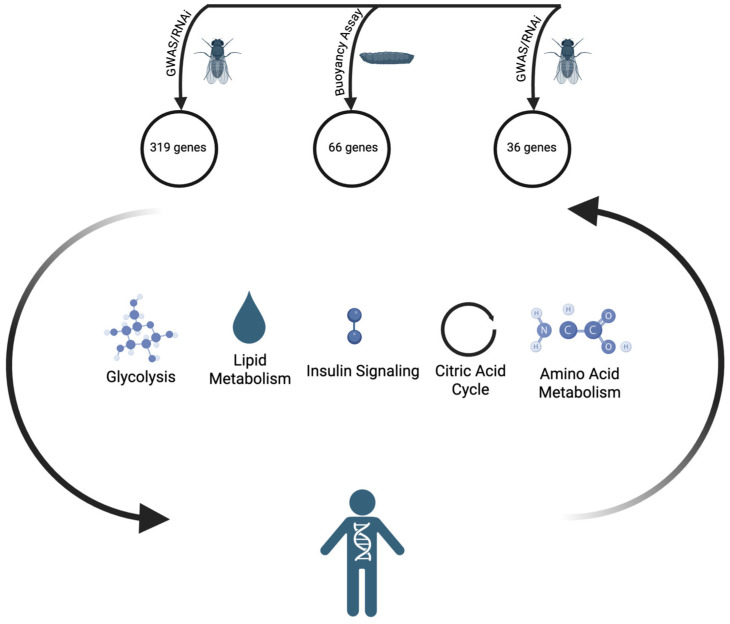
Genetic screens in *Drosophila* identify candidate human obesity genes. The three genetic screens for which results are detailed in Table 1 are schematized here. Two screens in adults and one screen in larvae generated lists of candidate genes regulating fat storage. Some known and predicted functional categories corresponding to these genes are illustrated in the center. The human figure below illustrates how information about the *Drosophila* genes can be used to inform interpretation of human obesity GWAS studies and how human obesity GWAS studies can be used to guide *Drosophila* genetic screens.

Whereas GWAS in human populations relies on spontaneous or pre-existing mutations/polymorphisms in individuals of diverse genetic backgrounds, *Drosophila* additionally offers the ability to examine the effects of experimentally induced mutations or manipulation of gene function (e.g., via RNA interference, also called RNAi) in large numbers of otherwise genetically identical individuals. Accordingly, six genetic screens have been performed focused on fat storage in *Drosophila* [27,28,29,30,31,32] (see Figure 1). One screen (often overlooked in the subsequent literature) was performed in larvae, using a collection of homozygous viable insertion mutants [31], meaning that null alleles of essential genes were not tested. Additionally, the mutants screened did not cover all non-essential genes. To identify fat mutants, Reis et al. developed a robust buoyancy-based assay to indirectly measure body fat content in the larval stages [31]. In an aqueous solution of fixed density, larvae with higher fat content will float more quickly and to a greater extent than larvae with lower fat content. 66 mutants accumulated more fat and represented genes with clear human counterparts [31]. The other five screens were performed in adult flies and relied on RNAi to inhibit gene expression. Only one of these adult RNAi screens was, like the larval screen, unbiased, meaning there was no attempt to target specific genes; the RNAi lines used in this screen targeted ~75% of the protein-coding genes in the fly genome [30]. Hedgehog signaling emerged from the unbiased RNAi screen as a common pathway affected by RNAi lines causing obesity; the hedgehog pathway is also known to inhibit fat formation in mice [33]. Baumbach et al. used an RNAi library enriched for orthologs of human genes [29], and again found some fly genes with mammalian homologs implicated in fat storage. For example, *TCF7L2* (*pangolin* in *Drosophila*) knockout mice are leaner and have improved glucose tolerance [34]. Baranski et al. used RNAi lines targeting fly genes that were homologous to candidate human obesity genes identified by GWAS [28]. Trinh et al. used RNAi lines targeting 1748 genes expressed in the nervous system [32]. In this study, targeted genes that had human orthologs linked to obesity via GWAS were given special consideration during the screening [32]. Similarly, with the goal of identifying GWAS-associated genes with anti-obesity functions, Agrawal et al. tested the requirement of 24 fly orthologs of such GWAS-linked genes and identified four genes that were required in adult male flies to maintain proper TAG levels [27].

A single gene (*ATP6AP2*) common to the hits from Pospisilik et al. [30] and Baumbach et al. [29] represents the only example of overlap between any of the adult RNAi screens in terms of genes that increase fat when inhibited. Studies of mammalian ATP6AP2 (also known as the (pro)renin receptor) implicate it in the regulation of gluconeogenesis [35] and linking glycolysis and the TCA cycle [36]. The two unbiased screens yielded as many common hits (one, *Arc1*) as the two RNAi screens, despite the differences in developmental stage (adult versus larvae) and method of gene perturbation (RNAi versus insertional mutagenesis). In terms of identifying conserved genes that might explain human GWAS results, the Baranski et al. [28] study that focused on specific genes based on human GWAS data was not much more effective than the unbiased screens: of the high-fat hits from Reis et al. [31] and Pospisilik et al. [30] 18 genes and 23 genes, respectively, had human counterparts within candidate GWAS loci for obesity, BMI, or type II diabetes, whereas Baranski et al. [28] found 29 (Table 1).

**Table 1:** Fly genes identified in genetic screens using the phenotype of increased organismal lipid levels and having human orthologs implicated in human metabolism via GWAS. For each study, the fly genes that caused high-fat phenotypes when targeted were analyzed using FlyMine [37] to identify human orthologs. Among these human orthologs, those that are linked via GWAS to human obesity, BMI, or diabetes were then identified using HumanMine [38]. The fly genes shown are only those with such GWAS-linked human orthologs.

Fly Gene	Human Gene
**Reis 2010 [31] (larval buoyancy, viable homozygous P-element insertions)**	
clt	ACHE
boi	DCC, NEO1, ROBO2
Gdi	GDI2
lilli	AFF3
trx	KMT2A
Fas2	NCAM1, NCAM2
NFAT	NFATC1, NFATC2, NFATC5
Fur1	PCSK6
Eip75B	PPARA, PPARD, PPARG, RARB, THRA
shep	RBMS1, RBMS3
Glut1	SLC2A2
tmod	TMOD1, LMOD1
msn	TNIK, MINK1
kibra	WWC1
hdc	HECA
trn	LINGO1, LINGO2, RTN4RL1, ELFN1
jim	ZNF257, ZNF713
jing	AEBP2
**Pospisilik 2010 [30] (adult triglyceride levels, unbiased RNAi collection)**	
CHES-1-like	FOXN3
Ets96B	ETV5
ttv	EXT1
eya	EYA1, EYA2
CG17026	IMPA2
CG34404	MCC
DIP-alpha	NCAM1, NCAM2, CHL1, IGSF9B
RabX6	RAB1A
RasGAP1	RASA2
CG8654	SLC22A3, SLC22A2, SLC22A11
org-1	TBX15, MGA
CG6689	ZNF229, ZNF268, ZNF585B
Ostgamma	TUSC3
mim	MTSS1
Vha100-5	ATP6V0A2, (pro)renin receptor
Odc1	ODC1
Su(fu)	SUFU
kmr	PLEKHA5
didum	MYO5C
Kif3C	KIF17
foi	SLC39A8
Zir	DOCK8
CG30486	CRISPLD2
**Baranski 2018 [28] (adult triglyceride levels, RNAi targeting human GWAS candidates)**	
BCL7-like	BCL7A
emp	SCARB2
fne	ELAVL4
Egfr	ERBB3, ERBB4
foxo	FOXO3
trh	NPAS1
park	PRKN
sv	PAX4, PAX5
CLIP-190	CLIP1
Ets98B	SPI1
pan	TCF7L2
Nrx-1	NRXN3, NRXN1, NRXN2
TTLL4A	TTLL4
CG1695	SGSM2
Aos1	SAE1
cert	CERT1
CG42458	RALY
Su(Tpl)	ELL2
Cluap1	CLUAP1
su(sable)	ZC3H4
spidey	HSD17B12
stumps	BANK1
CG4622	ZCCHC8
Nna1	AGBL2
mof	KAT8
Sec16	SEC16B
Pdi	PDILT
CG10465	KCTD13
CG4945	SBK1

Two other types of genetic screens, targeting neuronal activity instead of genes, have identified specific populations of neurons in the brain contributing to metabolic dysfunction in the adult [39] and larval stages of the fly [40]. Through the use of neuronal silencing and hyperactivation tools, 350 different subsets of neurons were manipulated in adults, and silencing of two of these lines, *c673a-Gal4* and *Fru-Gal4*, resulted in defects in adult fat storage [39]. These obesity phenotypes resulted in part from increased food intake. While the mechanistic details remain unclear, the results of this study suggest that the fly brain can assess levels of stored fat and alter gene expression in various other tissues to change behaviors and metabolism, preserving organismal energy homeostasis. A neuronal screen using the density-based approach identified groups of neurons in the larval brain that regulate body fat [40]. Mosher et al. identified E347 neurons as important regulators of fat storage: when E347 neurons were silenced, larvae became fat, and when stimulated, they became lean. Arc1, a conserved neuronal plasticity protein involved in learning and memory, was previously identified as a regulator of fat in two independent screens [30,31] and was found to be required for E347 neuronal regulation of body fat [40]. The identification of *Arc1* in independent, unbiased approaches further highlights the important but poorly understood connection between brain function and energy storage. It was later shown that neuronal *Arc* can form virus-like capsids, packaging its own mRNA, and travel via extracellular vesicles across neuromuscular junctions [41]. This ability to form viral-like capsids is conserved in mammals [42]. It remains to be discovered whether this mechanism plays a role in intercellular metabolic signaling.

In a growing fly, demands for nutrient and energy usage, production, and availability fluctuate throughout the lifespan (reviewed in [43]). Another added benefit of the fly model is the availability of assays that easily test for fat levels at different stages of development, allowing for investigations of energy homeostasis in the context of a growing organism and throughout the lifespan. At the larval stage, animals are dedicated to feeding to promote growth. This focus on converting dietary energy into fuel for growth represents a powerful model to study regulation of metabolism during development. Studies exploiting this model revealed that a switch in gene expression during embryogenesis drives a switch to aerobic glycolysis that sustains the accumulation of biomass required for larval growth, reminiscent of the Warburg effect in cancer cells [44,45]. The *Drosophila* homolog of the estrogen-related receptor (ERR) family of nuclear receptors drives this metabolic switch, which includes upregulation of TCA cycle and electron transport gene expression, driving accumulation of metabolites like citrate and α-ketoglutarate [44,45]. Work on germline stem cells (GSC) revealed a response to nutrient changes [46]. When fed a protein-rich diet, GSCs develop three times faster than on a protein-free diet. These effects are accompanied by a reduction in egg laying in the protein-free diet [46]. Crosstalk with fat storage cells also contributes to the regulation of female GSCs through adipocyte insulin signaling and amino acid sensing [47,48,49].

These examples highlight how powerful *Drosophila* continues to be as a model for human disease, uncovering new pathways to explore the basis of metabolic dysfunction in the context of an intact, complex organism while using many established genetic tools combined with behavioral and -omics approaches. The application of new tools has further added to the strengths flies offer as a model organism. *Drosophila* has been at the forefront of CRISPR-Cas-based manipulation of gene function in metazoan systems. While no CRISPR-based genetic screen for metabolism-related phenotypes has yet been reported, CRISPR-Cas9-mediated gene modification has been used to dissect gene function in fat/lipid storage or related phenotypes [50,51,52,53]. Single-cell RNA-sequencing technologies enabled the creation of reference atlases of transcriptomic tissue-specific signatures. The Fly Cell Atlas gathered experts from 40 laboratories across the world to create a resource for studies of gene function at the single-cell resolution [54]. Tissues analyzed in this study include the fat body, the gut, and insulin-producing cells. Importantly, these experiments were carried out in male and female tissues to allow for analyses of sexual dimorphism. These resources provide a uniform reference for single-cell transcriptomic maps of adult, metabolic *Drosophila* tissues. Leveraging the power of metabolomics to reveal changes in metabolism, the FlyMet database (http://flymet.org/) is conceptually similar to FlyAtlas but for metabolites.

Another set of tools that advanced studies in the field are FRET-based metabolic sensors that report on conformational changes induced by metabolite binding [55]. In a recent study [56], glucose [57,58], citrate [56], and lactate [56] sensors were used to measure metabolic and physiological effects of intestinal carbohydrate metabolism. Using tissue-specific midgut drivers, the group was able to identify sex differences in the concentration of these metabolites in enterocytes, with glucose being higher in males and both lactate and citrate being higher in females [56]. The availability and diversity of these resources highlight the collaborative nature and cutting-edge methodologies of the *Drosophila* research community.

## 3. Fat Flies: A Well-Controlled Animal Model for the Study of Gene-Diet Interactions

The interactions between the effects of genetic background and of environment on metabolic homeostasis are complex and represent an active area of research. Diet is a major environmental factor in metabolism. Just like genetic models of obesity can be characterized in *Drosophila*, diet-induced models recapitulate mammalian phenotypes. When fed a high-fat diet, flies accumulate excess fat and exhibit defects in heart function [59]. The diet-induced increase in triglycerides and dysregulation of insulin levels were linked to the TOR signaling pathway [59]. Furthermore, reduction in insulin-TOR signaling can ameliorate high-fat diet-induced obesity and cardiac dysfunction [59]. Drosophila larvae fed a high-sugar diet recapitulate important aspects of type 2 diabetes (T2D), including hyperglycemia, insulin resistance, and excess fat [60], providing a robust model to study the interplay between genes and environment that results in T2D. Musselman et al. used gene expression analysis to characterize this diet-induced T2D model and identify its key players at the larval stage. *Drosophila* insulin-like peptides were upregulated, similar to changes seen in adults fed a high-sugar diet [60].

Insulin and leptin signaling pathways regulate each other [61], and leptin signaling connects feeding with satiety. Human *leptin* is produced primarily by adipocytes. In *Drosophila*, two proteins, Upd1 and Upd2, play analogous roles to leptin, one secreted from *Drosophila* fat storage cells when flies are well fed [62,63], and the other produced in the brains of fed animals [62]. Remarkably, human *leptin* expressed in *Drosophila* is able to bind the receptors that normally bind Upd1 and Upd2 [62,63] demonstrating overall conservation of this signaling pathway.

To learn more about how animals maintain energy balance despite dietary changes, several studies manipulated diets in existing *Drosophila* lines of diverse genetic backgrounds. Diet manipulations ranged from an excess of individual (or specific combinations of) macronutrients to full restriction, i.e., starvation. One study monitored multiple phenotypes related to metabolic homeostasis in a collection of 146 natural *Drosophila* isolates [64]. Flies fed varied diets display distinct phenotypic responses to the same diet, emphasizing how gene-diet interactions are highly pervasive. Two more recent studies used ~200 inbred strains of distinct backgrounds and, after identifying variable diet responses, mapped SNPs that correlated with starvation resistance [65] or survival on different diets [66]. One such gene in which a polymorphism confers starvation resistance encodes a likely conserved glucose transporter that the authors showed to be required for survival on a high-carbohydrate diet [65]. Strikingly, in the other study, a different sugar transporter was required for survival on a sugar-free diet [66]. Finally, one study analyzed how four different “mitotypes” (distinct mitochondrial genomes found in *Drosophila* lines) responded to diets varying in protein and carbohydrate content [67]. Mitochondrial genetic background strongly influenced the metabolic response, including in some cases a relative increase in physical activity. Collectively, these studies illustrate how *Drosophila* as a genetic model system can be exploited to rapidly identify gene-diet interactions.

Deliberate dietary changes can also be used to ameliorate obesity phenotypes, providing a window into which pathways and metabolites are of critical importance. *Drosophila* studies have also exploited this approach, revealing that, beyond simply counting calories, manipulating the sources of dietary calories to match a specific organismal metabolome can partially restore multiple aspects of energy homeostasis. For example, mutants of a fatty acid desaturase die as larvae, but supplementation with fatty acids restored viability to adulthood as well as mating and fertility [68]. Similarly, a study of endoribonuclease *Arlr* found that it is required to maintain lipids in lipid droplets as adult flies age and then restored lipid droplet sizes and numbers with a high-fat or high-sugar diet [69]. In a more complex metabolic scenario, one study examined larvae depleted for the RNA-binding protein *Spen*, which acts in an autonomous manner in fat storage cells to regulate mobilization of energy [70]. Based on careful prior metabolomic analysis of the Spen-depleted larvae, the authors supplemented the diet in ways designed to provide more of the energy sources that the mutant larvae appeared to depend upon [71]. Some of these “custom diets” were able to improve longevity and speed up development at both the adult and larval stages. The idea that there are complex changes in organismal metabolism in flies harboring a single genetic lesion is also clearly illustrated by studies of larvae in which a key metabolic enzyme, lactate dehydrogenase, was mutated [72]. Whereas one expected larval development to be severely compromised by the loss of a critical step in a metabolic program that efficiently converts nutrients into biomass, instead the mutant larvae grew at normal rates. Excess production of glycerol-3-phosphate allowed the mutant larvae to compensate for the loss of lactate. Crucial to these studies was sensitive and broad quantification of metabolites (metabolomics). Metabolomics in *Drosophila* has emerged as a powerful tool to understand organismal energy homeostasis [73].

Determining the contribution of, or measuring the response to, specific dietary components requires being able to specifically manipulate the composition of the diet. A simple way to modify the diet involves changing the ratios of the primary ingredients in rich (undefined) fly food, which (depending on the recipe) typically include protein-rich dry yeast, cornmeal, malt extract, and corn syrup. Modifying the proportion of yeast is an easy way to adjust the relative protein content. Alternatively, protein in the form of tryptone or peptone can be added to rich food, such as was performed recently to discover that a peptide hormone secreted from the gut signals to enteric neurons to produce a neuropeptide that controls satiety in response to a high-protein diet [74]. However, more precisely altering diet composition requires synthetic or holidic diets in which the concentration of each macronutrient is more precisely known [75,76,77,78,79,80]. Since larval energy storage dynamics change during development, an important factor to consider for larval studies is how diet affects developmental timing. Ideally, overall timing of development is equivalent between different diets so that changes in energy balance and metabolism are direct effects of diet rather than indirect effects of altered development. Recent work found that larval gustatory organs allow them to discriminate between foods based on texture/hardness [81], which is a variable that should now also be considered.

One variable that has only recently been studied in detail is sex: metabolism in males and females is fundamentally different, in both humans and flies (reviewed here [82]). Hormones that control the development of sex-specific anatomical differences have some effects on metabolic pathways, but what has been less studied is how the fat storage tissues themselves in males and females are intrinsically different as a result of their chromosomal composition. Sex determination in flies involves alternative splicing of transcripts that encode regulators of anatomical development as well as sex-specific behaviors such as courtship [83]. As in mammals, fly metabolism is sex-dimorphic [82]. The gene *spenito* (*nito*) is closely related to *split ends* (*spen*), which was implicated in fat storage via the larval buoyancy-based unbiased screen [31]. Follow-up studies showed that Nito antagonizes Spen’s role in fat storage [70]. Spen’s role in fat storage is conserved in mammals [70], and human *SPEN* mutations are found in very obese children with autism spectrum disorder [84]. Around the same time, Nito was found to function in the alternative splicing that drives sex determination [85,86,87,88,89]. Recent studies found that Nito-dependent sex-specific splicing of these regulators also occurs in the fat storage cells of *Drosophila* larvae and is accompanied by distinct patterns of gene expression of metabolic enzymes, which correlates with differences in fat storage between male and female larvae [90]. Experimentally “feminizing” the fat storage cells by expressing the female splice isoform of one of the master regulators in an otherwise male larvae drives female-like gene expression and fat storage [90]. Future studies will be needed to understand the mechanistic details of these metabolic dimorphisms and their biological implications.

## 4. Conclusions and Outlook

Metabolism at the cellular level is complex and is even more so at the organismal level. Challenges in addressing the rise in human obesity reflect this complexity and highlight the need for experimental systems with robust genetic tools and precise control of environmental conditions. *Drosophila* provides a powerful system to study metabolism at the organismal level and thereby to reveal potential causes of human obesity and other metabolic disorders. In addition to serving as a tool to study conserved genes linked to obesity by human GWAS, the tractability of the *Drosophila* system allows unbiased, genome-wide genetic screens to identify new roles for genes not previously linked to metabolism. Tissue-specific manipulation of gene function has revealed signaling pathways between tissues and organs that regulate energy balance. By facilitating the discovery of such pathways, *Drosophila* research identified new potential targets for the development of possible therapeutics and the foundation for new clinical directions. Key topics for future research include better understanding how the brain regulates energy storage and expenditure in peripheral tissues and the contribution of the functions of different organs to the metabolism of the whole organism, especially in the context of changes such as development and aging.

## Data Availability

No new data were created or analyzed in this study.
